# Hybrid electronic record: An error reduction strategy for diverse medical prescription formats

**DOI:** 10.4102/safp.v66i1.5845

**Published:** 2024-06-07

**Authors:** Carl-Heinz Kruse, Michelle T.D. Smith, Damian L. Clarke

**Affiliations:** 1 Department of Ophthalmology, School of Clinical Medicine, University of KwaZulu-Natal, Durban, South Africa; 2 Department of Surgery, School of Clinical Medicine, University of KwaZulu-Natal, Durban, South Africa; 3 Department of Surgery, School of Clinical Medicine, University of the Witwatersrand, Johannesburg, South Africa

**Keywords:** human error, error rate, prescription, prescribing, medication, electronic health record, electronic medical record, handwritten

## Abstract

**Background:**

This project is part of a broader effort to develop a new electronic registry for ophthalmology in the KwaZulu-Natal (KZN) province in South Africa. The registry should include a clinical decision support system that reduces the potential for human error and should be applicable for our diversity of hospitals, whether electronic health record (EHR) or paper-based.

**Methods:**

Post-operative prescriptions of consecutive cataract surgery discharges were included for 2019 and 2020. Comparisons were facilitated by the four chosen state hospitals in KZN each having a different system for prescribing medications: Electronic, tick sheet, ink stamp and handwritten health records. Error types were compared to hospital systems to identify easily-correctable errors. Potential error remedies were sought by a four-step process.

**Results:**

There were 1307 individual errors in 1661 prescriptions, categorised into 20 error types. Increasing levels of technology did not decrease error rates but did decrease the variety of error types. High technology scripts had the most errors but when easily correctable errors were removed, EHRs had the lowest error rates and handwritten the highest.

**Conclusion:**

Increasing technology, by itself, does not seem to reduce prescription error. Technology does, however, seem to decrease the variability of potential error types, which make many of the errors simpler to correct.

**Contribution:**

Regular audits are an effective tool to greatly reduce prescription errors, and the higher the technology level, the more effective these audit interventions become. This advantage can be transferred to paper-based notes by utilising a hybrid electronic registry to print the formal medical record.

## Introduction

Human error has been recognised as being ubiquitous in modern health care systems and in contributing significantly to both morbidity and mortality. The publication of ‘*To err is human*’ at the beginning of the millennium,^1^ brought a lot of interest in strategies to reduce both the frequency and impact of health-related human error.^2,3^ This has become ever more important with the growing criminalisation of human error in medical practice.^2^

Electronic health record (EHR) systems offer the potential to embed error reduction strategies in their interfaces. There have been attempts in the South African environment to use the so-called clinical decision support systems (CDSS) in a hybrid electronic medical registry.^4,5^ An example of such electronic error reduction measures would be, a flashing warning of possible hypovolaemic shock if the entered blood pressure is below a set value. The data-entering clinician then has to respond by either taking corrective measures or stating that shock is not present. In effect, the system forces the clinician to reassess his assessment.

As part of this project, this group has reviewed the impact of various prescription formats and its effect on prescription errors in the ophthalmology service and has published the findings.^6^ This initial project found that merely increasing the level of technology did not produce the expected reduction in prescription error rates: Electronic and ink stamp-based prescriptions contained significantly more errors than the hand-written and tick sheet type prescriptions. However, the pattern of these errors seemed more consistent than in less technologically advanced prescription strategies. This study follows up on that previous work and attempts to integrate these earlier findings into a more effective CDSS designed to limit human error in prescribing in our wide spectrum of health facilities.

### Aim and objectives

This project is part of a broader effort to develop a new electronic registry and medical record for ophthalmology across the training platform in the KwaZulu-Natal (KZN) province in South Africa. The objectives for this initiative include capturing data for research purposes, providing the first eye-care electronic record system in the region and developing a CDSS that reduces the potential for human error. This reduction in error should be applicable and useful for the diversity of health facilities in our province, whether EHR, paper-based or other.

## Research methods and design

### Study design

This study was a retrospective analysis of errors made in prescribing medication in four different formats. We chose cataract surgery discharge medication as the common measure for two reasons: The chosen facilities are all state hospitals in KZN province and included Grey’s Hospital (handwritten), McCord’s Hospital (tick-sheet), Northdale Hospital (ink stamp) and Inkosi Albert Luthuli Hospital (electronic prescriptions). They all had this surgery in common, and because they all fall under the same university and rotating clinical department, the surgery and peri-operative protocols should be performed in a standardised manner.

In this study, potential remedies to the errors discovered were sought by dividing the process into four steps:

- Evaluation of each hospital’s prescribing system in an attempt to find possible causative systematic obstacles to accurate prescribing.
- Looking at the differences in types of error class for each prescribing technique and attempting to match them to the obstacles identified.
- Identification of easily correctable errors by comparing the errors made, to the existing systems available in the facilities.
- Using these results to inform the creation of a bespoke database for medical recording and dispensing while minimising the potential for errors.

Errors were considered easily correctable if they fulfilled all of the following three conditions:

- Minimal training: Retraining clinicians on changes made should be easily restricted to a once-off instruction, for example, ‘We have made this new box for you to fill in the date’. Training was not considered minimal if it required more than one step or action, or more than one session.
- Rapid implementation: The (previously designed) intervention was considered rapidly correctable if it could be implemented within 1 week, for example have a new stamp made, programme a software change and identify a file storage area. The planning and design of this error-correcting mechanism was part of the aforementioned ‘remedy discovery’ part of this study and was not counted as part of this week.
- Fixed changes: It is a natural response to fall back into old habits. The aim of the error-correcting amendments would be to guide the prescribers without them requiring constant reminders. Amendments made should not be easily reversable or avoidable without conscious effort on the part of the end-user.

### Setting and study population

Comparisons were facilitated by each of the four hospitals having a different system for prescribing medications: electronic, tick sheet, ink stamp and handwritten health records.

#### Electronic health record

With a comprehensive EHR, Inkosi Albert Luthuli Central Hospital is a quaternary-level facility and functions as an almost entirely paperless entity. The system includes an electronic ‘Scripting Module’ and has the greatest range of available medications of the four hospitals. The prescription structure is modifiable and changes to the software can be requested by individual departments from the in-house central information technology (IT) centre.

#### Ink stamp prescriptions

Northdale Hospital is a busy district level hospital but has instituted an ink stamp system for the most common conditions, including elective post-operative discharge medication. Because of the inflexibility of the stamp system, it is expected of the clinician to manually include missing information such as route (eye) and laterality (left/right), add patient-specific medication that is not included on the stamps and manually delete standard stamp medications that are contraindicated or unnecessary for the particular patient. The patient folders from the various clinics in the facility, which include the stamped paper prescriptions and doctors’ notes, are collected and stored in a central hospital registry.

#### Tick-sheet prescriptions

McCord’s Provincial Eye Hospital is a secondary level facility and had developed its own bespoke prescription tick sheet, which contains almost all the commonly used medications in the relevant speciality. Patient details are added to this prescription by pre-printed sticker, but the clinician still needs to write their name, date and less common medications that have not been pre-populated on the tick sheet. These tick sheet scripts remain in the patient files.

#### Handwritten prescriptions

Despite being a tertiary academic institution, Grey’s Hospital uses an entirely handwritten prescription format – only the date is stamped onto the relevant document before the patient sees the clinician. Although most speciality departments store the patient files in the central hospital registry, the clinic that was the source for the data in this study, stores duplicate patient records separately.

### Sampling strategy

Records of all patients were included, irrespective of age, sex and race. No sampling strategy was used; post-operative prescriptions of consecutive cataract surgery discharges were obtained for 2019 and 2020. Lists of cataract surgery cases were retrieved from theatre records, and original patient files were obtained and used as source documents. If the entire patient record could not be found, the patient was excluded from the study. If the file could be found but the relevant prescription was missing, this was categorised as an error of ‘script not found’.

### Data collection

**FIGURE 1:**
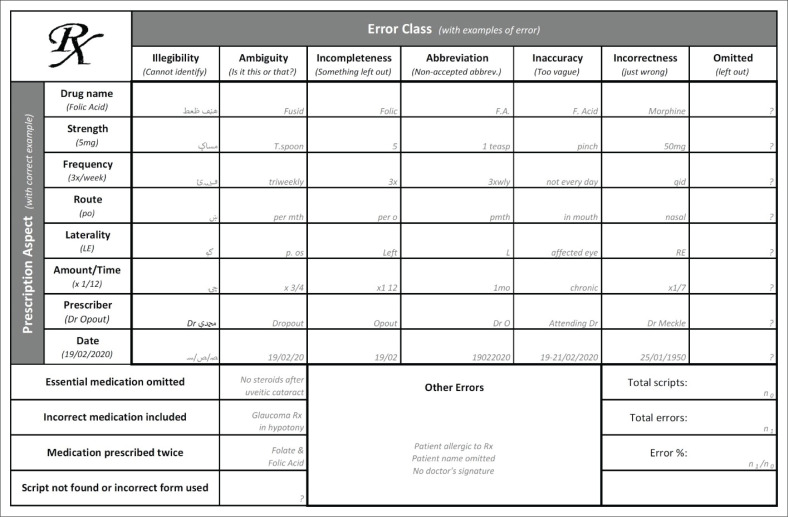
Our prescription error data collection tool with an example (greyed-out) in each cell.

Figure 1 shows the data collection tool we developed and used for this study. Evaluation of errors was done by all authors according to prescribed national scripting norms^7^ and were classified and presented as ‘prescription aspect’ (rows), ‘error class’ (columns), ‘general errors’ and ‘other’.

### Data analysis

All data were categorised and tabled in a flat spreadsheet. R Studio™ (R Foundation for Statistical Computing) was used for all statistical calculations. All variables were expressed as frequencies and percentages. Handwritten scripting errors were used as the baseline against which all other prescription types were measured. The frequencies of error classes across all prescription aspects were compared to the errors of the ink stamp, tick-sheet and the electronic systems using the Chi-square test with an alpha level of 0.05. Odds ratios (ORs)were calculated for all comparisons.^6^

### Ethical considerations

Prior approval was obtained from the research ethics committee of the University of KwaZulu-Natal (Study approval number: BREC/00002077/2020) as well as all four health facilities and the KwaZulu-Natal provincial Department of Health.

This article does not contain any studies involving physical human participants, only retrospective patient records. No identifying data were collected from source documents and identifying data are not used in publication or public distribution. All source documents are legal patient records and remain the property of the relevant hospital.

## Results

Across the four hospital facilities, a total of 1661 scripts were reviewed and 1307 individual errors were found (Table 1). Enumerating the errors in each prescription format found that, contrary to expectations, increasing technology did not reduce error rates. Ink stamp and electronic prescriptions contained significantly more errors than in the hand-written and tick sheet formats (*p* < 0.001).

**TABLE 1:** Prescription error types in handwritten versus other scripting formats (*n* = 1307).

Error type	Hand-written (*n* = 355)		Ink stamp (*n* = 488)					Tick-sheet (*n* = 331)					Electronic (*n* = 487)				
	*n*	%	*n*	%	*p*	OR	95% CI	*n*	%	*p*	OR	95% CI	*n*	%	*p*	OR	95% CI
Drug name	1	0.3	3	1.0	0.474	2.1	0.2–59	5	2.0	0.116	4.3	1–113	0	0.0	0.245	-	
Strength	5	1.0	4	1.0	0.432	0.6	0.1–2.4	3	1.0	0.429	0.6	0.1–2.5	**86**	**18.0**	< 0.001	15.0	6.5–42
Frequency	2	0.6	3	1.0	0.904	1.1	0.2–9.4	3	1.0	0.699	1.4	0.2–12	1	0.2	0.397	0.4	0.0–4.9
Route	8	2.0	**119**	**24.0**	< 0.001	14.0	7.1–31	1	0.3	0.015	0.1	0.0–0.7	0	0.0	< 0.001	-	-
Laterality	0	0.0	**116**	**24.0**	< 0.001	-	-	9	3.0	0.003	-	-	**248**	**51.0**	< 0.001	-	-
Amount/time	**30**	**9.0**	6	1.0	< 0.001	0.1	0.1–0.3	3	1.0	< 0.001	0.1	0.0–0.3	0	0.0	< 0.001	-	-
Prescriber	5	1.0	20	4.0	0.020	3.0	1.2–9.2	**55**	**17.0**	< 0.001	12.0	5.0–34	0	0.0	0.009	-	-
Date	7	2.0	1	0.2	0.010	0.1	0.0–0.7	7	2.0	0.916	0.9	0.3–2.8	0	0.0	0.002	-	-
Rx omitted	74	21.0	76	16.0	0.071	0.7	0.5–1.0	52	16.0	0.013	0.6	0.4–0.9	134	28.0	0.021	1.5	1.1–2.0
Wrong Rx	5	1.0	0	0.0	0.009	-	-	0	0.0	0.021	-	-	4	1.0	0.427	0.6	0.1–2.3
Double Rx	12	3.0	12	3.0	0.462	0.7	0.3–1.7	5	2.0	0.067	0.4	0.1–1.1	21	4.0	0.466	1.3	0.6–2.8
Script lost	25	7.0	**108**	**22.0**	< 0.001	3.8	2.4–6.1	11	3.0	0.010	0.4	0.1–0.8	0	0.0	< 0.001	-	-
Other	6	2.0	2	0.4	0.630	0.3	0.0–1.2	9	3.0	0.500	1.4	0.5–4.3	0	0.0	0.004	-	-

**Total errors**	**178**	**50.0**	**470**	**96.0**	**< 0.001**	**2.5**	**1.9–3.3**	**163**	**49.0**	**0.079**	**0.8**	**0.6–1.0**	**494**	**101.0**	**< 0.001**	**5.4**	**4.0–7.4**

Note: Data in bold are larger errors unique to only one or two facilities.

**FIGURE 2:**
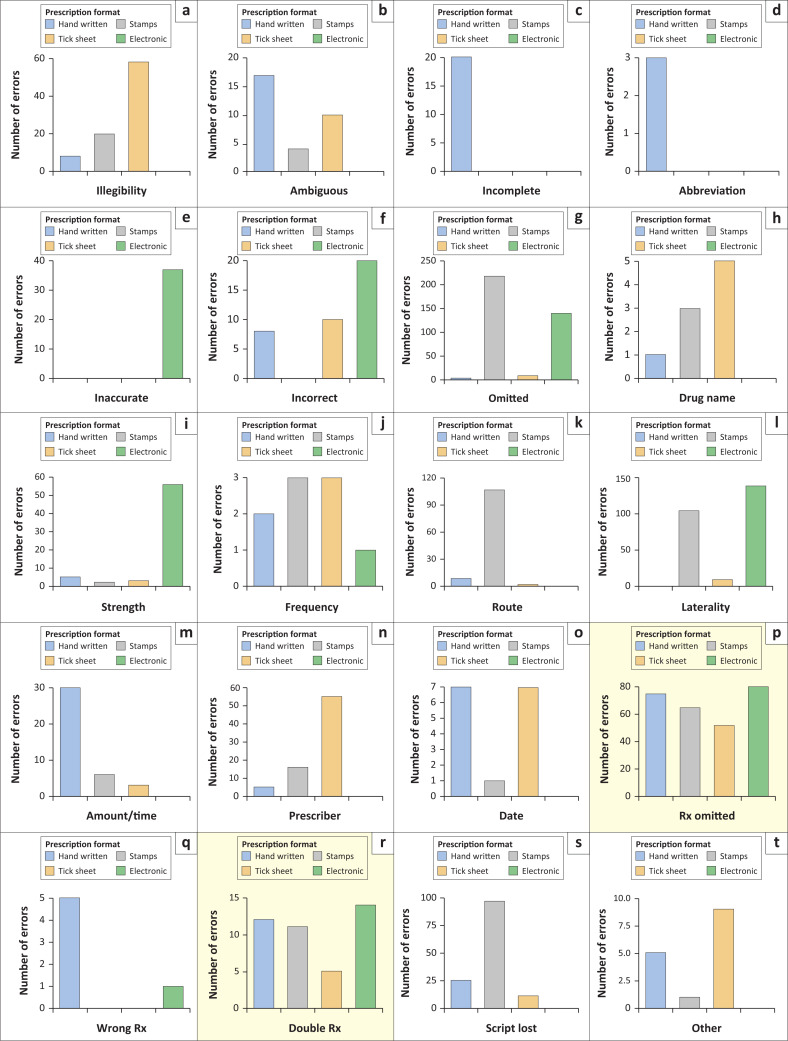
Prescription errors (*n* = 1307) by prescription format. Graphs p and r (highlighted) are the frequent error types common to all four prescription formats.

The 1307 errors could be categorised into 20 error types. As highlighted in Figure 2, only two of the 20 error types were universal to all four facilities and prescription formats: protocol-appropriate treatment that had been omitted (range: 52–134 errors) and incorrectly prescribing the same class of drug twice (range: 5–21 errors). Most other error classes were unique to only one or two of the four hospitals:

- The electronic system had the most errors and was particularly poor with regard to inaccurate, incorrect or omitted medication strength (*n* = 86) and laterality/which eye (*n* = 248).
- The ink stamp format performed particularly poorly in terms of lost prescriptions (*n* = 108), omitting the route the medication should be taken (*n* = 119) and laterality/which eye (*n* = 116).
- Tick sheet prescriptions did poorly in errors of illegibility (*n* = 23), omitting the name of the prescriber (*n* = 55), omitting the date (*n* = 7) and errors in drug name (*n* = 5).
- Handwritten prescriptions had high error rates in incomplete scripts (*n* = 20) and the duration/amount of the medication that should be taken (*n* = 30). Error types with fewer counts, but still particular to the handwritten format, included incorrect abbreviations (*n* = 3), prescription of the wrong medication (*n* = 5) and omitting the date (*n* = 7).

All results remained consistent when confounders such as patient complexity, pre-operative risk factors and facility level of care had been adjusted for.

**FIGURE 3:**
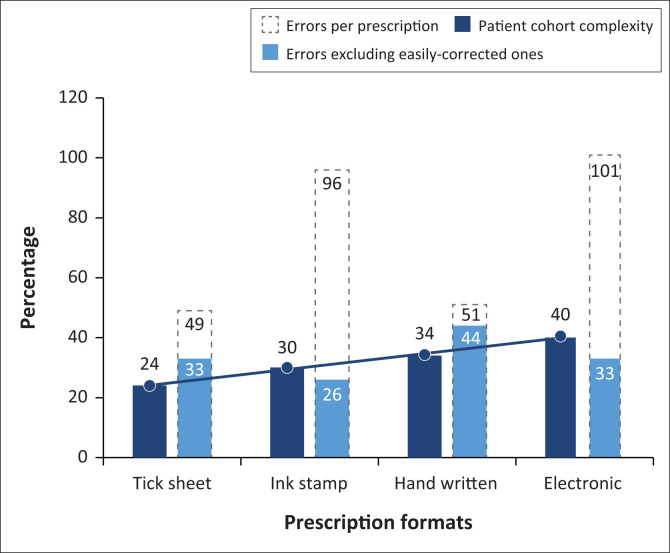
Number of errors per facility compared to facility patient cohort complexity, before and after removing all causes of easily-correctable errors. Patient cohort complexity had no effect on error rates.

The impact of correcting easily-correctable errors is shown in Figure 3.

### Electronic health record amendments

A large part of the errors in the EHR system arose because of the generic nature of the programming, which did not take factors such as laterality and dosage of ointments into account. As the same error is repeated with every prescription, it accounted for the vast majority of errors found. By simply adding the capability to determine ointment dosage and laterality to the software, electronic prescription error rates would decrease from 101% to 33%. (note that the error rate may be higher than 100% because more than one error could be made per prescription.)

The reason provided for not having fixed this issue was not that the system would not allow it, but rather that the IT department had not received feedback about the problem from the end-users since the inception of the system 20 years ago. Any such changes to this modern EHR system are relatively straightforward and rapidly implementable.

### Ink stamp amendments

The primary problem with the stamp form of prescribing was that the laterality (right/left) and route (eye) had been omitted from all the drops and ointments on the stamp. The stamps had originally been designed this way because this specific information would not be universal for all patients. The clinicians were expected to add the missing detail by hand, but we found that this was rarely done. One remedy would be to design stamps where any additional detail to be filled in is clearly demarcated by either a box outline or input line. This would serve as a visual prompt for the clinician to add the outstanding information.

The loss of patient files and prescriptions seems to be a particular issue where unrelated clinics store all notes in one large central paper archive. Keeping notes, or simply copies of notes, inside each relevant clinic seems to ameliorate this problem to a large extent. Together with a redesign of the stamps, the errors in the ink stamp prescription system would decrease from 96% to 26%.

### Tick sheet amendments

Illegibility and ambiguity of both the date and prescribers name are correctable by simply using date stamps upon patient entry and providing each clinician with a personalised identifying stamp with all his or her relevant details. Making these two changes would decrease the error rate in tick sheet prescriptions from 49% to 33%.

### Handwritten amendments

An attempt to implement all of the above-mentioned changes to the cohort of handwritten notes, would only reduce the prescription error rate by 7% (from 51% to 44%). Other error types in this group were very diverse and related to simple mistakes. Plain human error is typically more difficult to regulate than institutional or systemic errors.

After adjusting for easily-correctable mistakes, the handwritten prescriptions had the most errors of all prescribing formats (Figure 3).

## Discussion

The development of a hybrid system seems to also be an opportunity to introduce effective error reduction strategies into an institution. Since original health database input is electronic, the focus of error reduction should focus primarily on these systemic errors, such as errors in laterality and dosage. Forwarding this captured information to the official hospital medical record, whether electronic or paper-based, has the advantage of also forwarding the reduction in prescribing errors while catering for a wide range of hospitals and medical record types.

As ‘possibly the most unequal country on Earth’,^8^ health facilities in South Africa vary greatly with regard to the level of care, available funding and systems at their disposal. Developing an EHR system that can be rolled out across these multiple sites requires an innovative database structure, which complies with the modern legal requirements and integrates these diversities with the variations in hospital level, structure and culture. Hospitals already using an EHR system would find paper-based solutions unfeasible, while hospitals with paper-based records (the majority of state health facilities in South Africa) may lack the resources to adopt a fully fledged electronic system and the high level of IT support that would need to accompany it. The attempted solution is to incorporate both electronic and paper-based systems. The entry point to the registry is electronic but it produces a printed patient record that is entered into the patient file. In contrast to an EHR, in this hybrid system the printed notes function as the legal medical record. The hospitals with existing electronic EHR systems would follow a different route while using the same hybrid system – Instead of printing the data, the captured database information is pulled into the hospital’s formal EHR, either by direct copy-and-paste function or by automatic field population by means of electronic system integration.

This research is limited by individual and facility peculiarities that might not be relevant to other hospitals, even with the same scripting format. Further research in other facilities, whether African or other, would add insight to reducing errors in prescribing.

## Conclusion

Increasing technology, by itself, does not seem to reduce prescription error. Technology does, however, seem to decrease the variability of potential error types, which make many of the errors simpler to correct. Regular audits are an effective tool to greatly reduce prescription errors, and the higher the technology level, the more effective these audit interventions become. This advantage can be transferred to paper-based records by utilising a hybrid electronic registry to print the formal medical record.
